# The Hepato-Cardio-Renal Axis in Cirrhosis: Hemodynamic and Mechanistic Insights, Diagnostic Biomarkers, and Expanding Therapeutic Horizons

**DOI:** 10.15190/d.2025.17

**Published:** 2025-12-31

**Authors:** Srihitha Mesineni, Sona Arun, Ujwal Bose, Prajaa Vaiyamalai Jaisankar, Khushi Parag Shah, Abishek Harikumar

**Affiliations:** ^1^Faculty of Medicine, Tbilisi State Medical University, Tbilisi, Georgia

**Keywords:** Liver Cirrhosis, Cirrhotic Cardiomyopathy, Hepatorenal Syndrome, Hepatocardiorenal syndrome, Portal Hypertension, Splanchnic vasodilation, Hyperdynamic Circulation, Multi-Organ Failure.

## Abstract

Liver cirrhosis is increasingly recognized as a multisystemic disorder, profoundly impacting cardiac and renal function, giving rise to tightly coupled cirrhotic cardiomyopathy (CCM) and hepatorenal syndrome (HRS), and creating a vicious cycle of the hepato-cardio-renal axis. In this narrative review, we propose viewing CCM and HRS as triad dysfunction, driven by shared hemodynamic and molecular mechanisms linking portal hypertension, splanchnic vasodilation, hyperdynamic circulation, and systemic inflammation. We further synthesize the progression of intrahepatic resistance and splanchnic vasodilation to the hyperdynamic circuit, leading to triggers for CCM and HRS. Diagnostic biomarkers such as natriuretic peptides, troponins, neutrophil gelatinase-associated lipocalin (NGAL), kidney injury molecule-1 (KIM-1), along with emerging imaging techniques like artificial intelligence-cirrhosis-electrocardiogram (ACE), echocardiography, and point-of-care ultrasound (poCUS) are reviewed. We also discuss therapeutic horizons, while vasoconstrictors with albumin and diuretics remain crucial pharmacological care. Beyond drugs, transjugular intrahepatic portosystemic shunt (TIPS), liver transplantation, and lifestyle and nutritional modifications play a vital role. Persistent challenges arise due to constant reliance on the organ-centric approach, lack of true predictors of response, and risk of unmasking latent cardiac and renal vulnerability. Finally, future direction demands a paradigm shift to an integrated axis-aware, genetic, molecular, and cellular approach and the use of artificial intelligence to enable individualized risk stratification and improve long-term outcomes in cirrhotic patients with cardiorenal involvement.

## SUMMARY

*1. Introduction*

*2. Liver Cirrhosis as an Initiator of Multiorgan Dysfunction*

*3. The Pathophysiology of Hepato-Cardio-Renal Syndrome in Cirrhosis*

- *3.1 Development of Portal Hypertension to Systemic Vasodilation *

- *3.2 Establishment of Hyperdynamic Circulation*

- *3.3 Cirrhotic Cardiomyopathy (CCM): Molecular and Structural Alterations*

- *3.4 Hepatorenal Syndrome: Mechanism of Renal Circulatory Alteration*

4*. Diagnostic Considerations *

*5. Therapeutic horizons*

- *5.1 Pharmacological*

- *5.2 Non-pharmacological*

- *5.3 Lifestyle and nutritional interventions*

*6. Challenges and limitations*

*7. Future directions* *8. Conclusion*

## 1. Introduction

A global burden of 1.47 million deaths occurs due to liver cirrhosis, out of which nearly 1 million are directly attributable to the disease. Although traditionally viewed as a liver-specific condition, it also involves multi-systems, profoundly cardiovascular and renal systems, which has led to the discovery of “hepatocardiorenal syndrome,” a multi-organ syndrome^^1^^. Among the complications, cirrhotic cardiomyopathy (CCM), a heart disease, has alterations in both structure and function^^2^^. According to the 2019 CCM consortium, the prevalence of CCM is 55.7%, with diastolic dysfunction at 7.4% and systolic dysfunction at 53.3%^^3^^. Furthermore, renal involvement in cirrhosis, known as hepatorenal syndrome (HRS), is where patients with advanced liver disease develop acute kidney injury because of hemodynamic alterations ^^1^^. With an average probability of 18% and 39% in the first year and over 5 years, HRS manifests in 4% of hospitalized individuals with decompensated cirrhosis^^4^^. In a retrospective study conducted from 2016 to 2019, the rates of readmission and associated mortality of HRS within 30 days were 24.6% and 14.35%, respectively^^5^^. Moreover, without any treatment, the 90-day mortality rate can reach up to 75%^^6^^.The concept of the hepato-cardio-renal axis illustrates how liver dysfunction contributes to circulatory alterations, as well as neurohormonal activation, and systemic inflammation, which subsequently impair cardiac and renal function. Moreover, these mechanisms are interconnected, with cardiac dysfunction worsening renal failure and both acting as a catalyst to the advancement of liver disease, creating a vicious circle of multi-organ failure^^1^^. The hepato-cardio-renal axis is used as a conceptual axis based on- construct, giving us a broader perspective of how the recognized clinical syndromes, such as CCM and HRS, are interconnected, rather than replacing the existing clinical definitions of these syndromes^^1^^.There are still significant challenges, and gaps remain despite our growing understanding of these systems. HRS and intrinsic kidney disease are difficult to differentiate^^7^^. Cardiac diagnostics also present difficulties. In HRS, the high dependence on the serum creatinine level makes it unreliable^^8^^. The PoCUS protocol for Hepatorenal Syndrome-Acute Kidney Injury (HRS-AKI) from venous congestion remains unstandardized and insufficient^^9^^. For treatment of HRS-AKI, terlipressin combined with albumin shows a varied response^^10,11^^. Norepinephrine is less variably used, and TIPS further contributes to complexities involving cardiac dysfunction. There are global inequities in regions where the burden of cirrhosis is highest^^12,13^^. There is complete neglect regarding transplant-free survival and long-term renal recovery. Furthermore, very few studies dive deeper to assess the heart-kidney-liver axis simultaneously^^13,14^^.This review explains the changes in the hepato-cardio-renal axis in cirrhosis by analyzing pathophysiological mechanisms underlying hepatorenal syndrome and cirrhotic cardiomyopathy. It further provides a detailed overview of diagnostic biomarkers and current therapeutic options while highlighting challenges and gaps in current knowledge and stressing the need for novel strategies to improve prognosis and treatment in cirrhotic patients with cardiorenal involvement.

## 2. Liver Cirrhosis as an Initiator of Multiorgan Dysfunction

Various etiological causes have been linked to liver cirrhosis. Some of the most prevalent causes on a global scale are viral in origin- hepatitis B, accounting for 22.4% of cases, and hepatitis C, accounting for 26.8%. Non-viral causes have a wide etiology, where Alcoholic liver disease accounts for 24.9%, whereas only 9.2% were linked to non-alcoholic fatty liver disease^^15^^. The remaining 16.8% of cases are composed of other rarer causes such as

**Figure 1:**
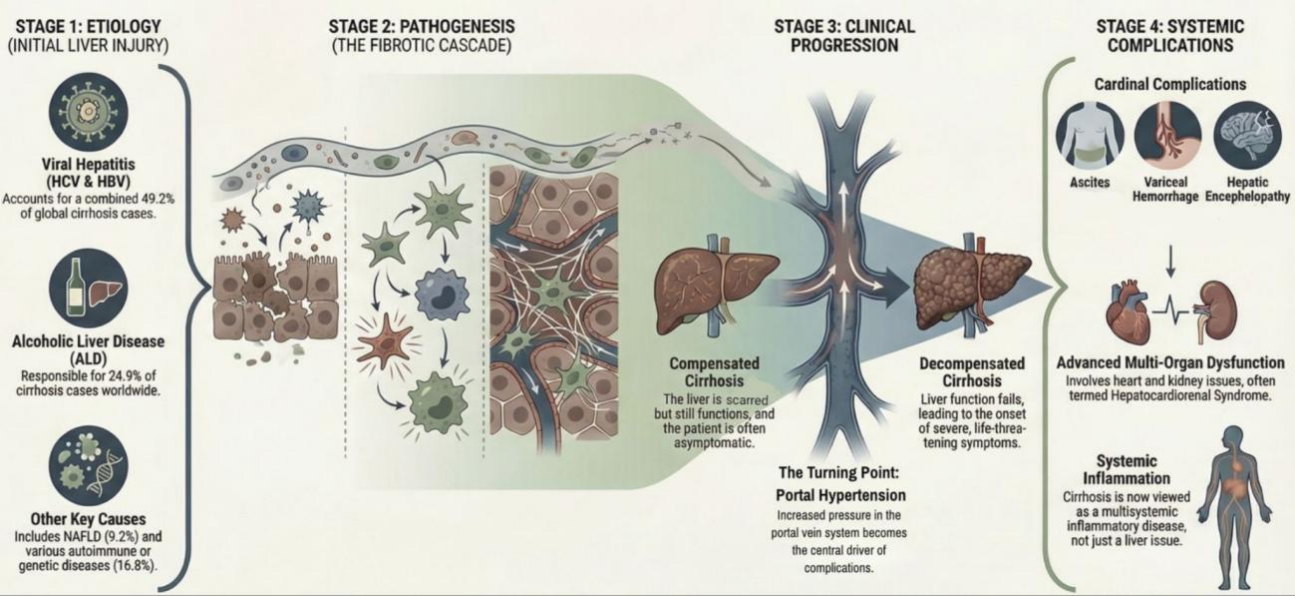
Liver cirrhosis: from etiological injury to systemic complications The above visual figure outlines the various causes of liver cirrhosis and its clinical progression from compensated to decompensated fibrosis, with rising portal hypertension driving systemic involvement, especially cardiac and renal. Alcoholic liver disease (ALD); Non-alcoholic fatty liver disease (NAFLD). Figure created by the authors using an AI-assisted visualization tool. All components are original and author generated. Not adapted from any previously published source.

autoimmune processes, storage diseases, toxic substances, drug-related, primary sclerosing cholangitis, primary biliary cholangitis, and even conditions such as Wilson’s disease and hemochromatosis^^15^^.

The process of hepatic fibrosis initiates with the activation of both stellate cells and Kupffer cells, along with the involvement of endothelial cells, damaged hepatocytes, and activated platelets. Inflammatory cells appear in response to damage and promote fibrosis by secreting cytokines. Over time, the formation of regenerative nodules is observed. Hepatic blood flow is then disrupted as a result^^16^^. Continuous activation of this process leads to excess extracellular matrix deposition, resulting in increased portal pressure and abnormal hepatocyte function^^17^^.

Furthermore, the natural course of cirrhosis evolves from compensated to decompensated states. It begins with the compensated phase, which is often asymptomatic, and the liver function is still intact. The progression to the decompensated state, with rising portal hypertension, acts as a central driver. This is followed by a cascade of hemodynamic, neurohormonal, and inflammatory alterations. These effects extend beyond the liver, initiating the onset of systemic involvement. It results in decompensated cirrhosis manifested through various pathophysiological pathways that give rise to cardinal complications of cirrhosis, such as ascites, variceal hemorrhage, and hepatic encephalopathy, which often lead to hospitalization. More advanced complications include hyperdynamic circulation, HRS, CCM, hepatopulmonary syndrome, and Porto-pulmonary hypertension^^18,19^^.

Portal hypertension and the resulting hyperdynamic circulation have been a primary focus. The narrative has now shifted to viewing liver cirrhosis as a multisystemic inflammation-driven disease. Systemic inflammation is a key factor in the extrahepatic complications observed in patients^^20^^. This highlights the true nature of liver cirrhosis as a complex multisystem disorder, in contrast to the earlier viewpoints. Among the extrahepatic conditions, renal and cardiac dysfunctions have particular importance as they are closely interlinked, forming the vicious cycle called hepatocardiorenal syndrome.**Figure 1**: provides us a visual overview of various etiological causes of liver cirrhosis and their clinical progression to systemic complications.

## 3. The Pathophysiology of Hepato-Cardio-Renal Syndrome in Cirrhosis

### 3.1 Development of Portal Hypertension to Systemic Vasodilation

The primary cause of the development of intrahepatic resistance is the alteration of the liver’s architecture. As the condition progresses, the liver Sinusoidal Endothelial Cells (SECs) lose their fenestrae by a process called sinusoidal capillarization. As fenestrae are lost, SECs acquire a basement membrane, markedly increasing sinusoidal resistance^^21^^.

Secondly, the SECs in cirrhotic liver produce less NO, due to reduced endothelial subtype of nitric oxide synthase (eNOS). Elevated arginase I decreases L-arginine, which is a main substrate for eNOS, further limiting intrahepatic NO synthesis. Moreover, thromboxane A2 (TXA2) reduces eNOS activity and NO bioavailability^^22^^.

The Kupffer cells-derived TXA2 activates thromboxane prostanoid receptors on SECs and hepatic stellate cells, promoting stellate-cell contraction and further elevating sinusoidal resistance^^23^^. Also, TXA2-mediated platelet activation predisposes to the formation of microthrombi and increased resistance to portal flow. HSCs also release endothelin 1, further constricting the intrahepatic microvasculature^^24-26^^. These mechanisms increase intrahepatic microvascular resistance, leading to portal hypertension.

In contrast, NO overproduction by eNOS and iNOS (inducible nitric oxide synthase) occurs in the splanchnic and systemic circulation, leading to vasodilation to overcome portal hypertension. Portosystemic shunting allows direct transfer of different gut bacterial endotoxins and pathogen-associated molecular patterns across the intestinal membrane, skipping the portal system^^27^^. These hemodynamic changes are related to an increased production of multiple inflammatory and endothelial mediators converging to markedly increased NO-dependent splanchnic vasodilation. As a result, a drop in splanchnic arterial resistance will reduce overall systemic circulatory resistance^^28,29^^.

Further mechanisms are summarized by the “Backward Flow” (BF) and “Forward Flow” (FF) theories of portal hypertension. Experimental models suggest that both BF and FF theories mechanisms together contribute to elevated portal pressure, respectively^^30,31^^.

Portal pressure is influenced by the interaction between portal inflow and intrahepatic vascular resistance^^32^^. Development of portal hypertension results in a subsequent systemic pressure drop, mainly due to profound vasodilation, driving a reduction in Systemic Venous Resistance (SVR), creating a pressure gradient towards systemic circulation from the portal system. This facilitates angiogenesis, where it forms collateral vessels connecting the portal system to systemic circulation^^33^^. Although shunting causes decompression of the portal system, reducing the hypertensive state, which reduces the risk of bleeding, it, however, exposes the systemic circulation to unmetabolized toxins^^33^^. In addition, hypoalbuminemia is seen in the blood due to reduced liver function, which diminishes plasma oncotic pressure, shifting fluid into the peritoneal cavity^^19^^.

Once portal hypertension and profound splanchnic vasodilation are established, the cardiovascular system, in response to the compensatory mechanism, results in the development of a hyperdynamic circulatory state.

### 3.2 Establishment of Hyperdynamic Circulation

SVR is drastically reduced by a small increase in systemic/splanchnic vessel radius, and in cirrhosis, marked splanchnic vasodilation lowers SVR well below the normal level^^34^^.

The fall in SVR decreases arterial pressure, activating SNS and renin-angiotensin-aldosterone System (RAAS), which increases heart rate, contractility, and stroke volume. The resulting rise in cardiac output partially offsets the reduced SVR, producing the hyperdynamic circulation observed in cirrhosis^^34-36^^. Inefficient myocardial compensation under stress may later contribute to cardiac dysfunction^^35^^.

Moreover, systemic-splanchnic vasodilation leads to pooling of blood and increased hydrostatic pressure within the splanchnic bed, reducing effective arterial blood volume, a phenomenon known as splanchnic steal^^37^^. As this condition worsens, hypoalbuminemia and hypervolemia account for reduced oncotic pressure in the luminal side. The osmotic pressure gradient is directed towards the peritoneal cavity, exacerbated by RAAS activation, as it overwhelms the drainage capacity of splanchnic lymphatics, leading to ascites formation^^35,38-40^^. As the condition progresses, the heart's ability to maintain the compensatory hyperdynamic circulation reduces; this mechanism will be explained in subsequent sections on CCM^^41^^.

In the early stages of cirrhosis, the body compensates for decreased SVR. The tipping point is when there is progressive vasodilation and neurohumoral overload along with cardiac dysfunction, leading to decompensation when it wanes and goes below 60 mmHg^^2,36^^.

Furthermore, neural studies show that afferent impulses from the vagus nerve increase the fos in the Tractus Solitarius Nucleus and paraventricular Nuclei, causing increased activity in central cardiovascular regulatory nuclei to cause hyperdynamic circulation in response to portal hypertension^^42^^.

Lastly, when these compensatory mechanisms fail, cardiac reserve becomes diminished. As a result, CCM emerges as a critical component in the hepato-cardio-renal continuum.

### 3.3 Cirrhotic Cardiomyopathy (CCM): Molecular and Structural Alterations

CCM is a cardiac dysfunction associated with cirrhosis, occurring in patients without an underlying primary heart abnormality. The condition is characterized by a blunted contractile response to stress, diastolic dysfunction, and electrophysiological disturbances. The severity of the condition is related to the degree of hepatic insufficiency^^43,44^^.

**Figure 2:**
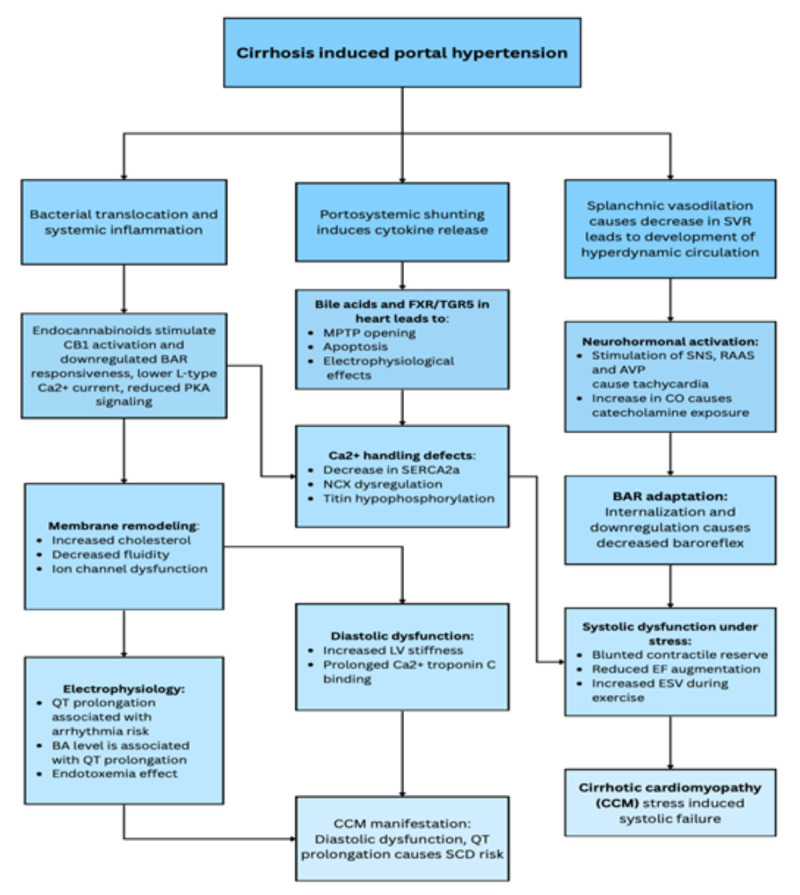
Pathophysiological mechanism of cardiac dysfunction induced by cirrhosis. Portal hypertension, being the major hemodynamic consequence in cirrhosis along with systemic inflammation, induces splanchnic vasodilation, giving rise to hyperdynamic circulation marked by elevated cardiac output but reduced systemic vascular resistance. These events trigger activation of sympathetic activity, RAAS upregulation, and prolonged catecholamine exposure, contributing to the downregulation of β-adrenergic receptor (BAR) and impaired contractile reserve. Additionally, overactivity of endocannabinoids, altered membrane fluidity, and defective calcium handling lead to disruption of excitation-contraction coupling. These changes impose myocardial remodeling, fibrosis, and diastolic stiffness, which result in the elevation of left ventricular filling pressures. Ion dysfunction and endotoxemia further promote QT prolongation and arrhythmogenic risk. All these processes collectively contribute to stress-induced systolic failure and increase the susceptibility to arrhythmias, summarizing the clinical course of CCM^44,46^. This figure is created using canva.

The direct negative inotropic effect of the released vasodilators like NO, Carbon monoxide, and endocannabinoids on the heart contributes to the development of advanced left ventricular diastolic dysfunction (LVDD), especially in cirrhotic patients with ascites compared to the ones without it^^43-45^^. **Figure 2** below provides a visual summary of the pathophysiological mechanism of cardiac dysfunction in cirrhosis.

#### 3.3.1 Cellular and Autonomic Dysfunction

In cirrhosis, reduced plasma membrane fluidity and altered lipid raft architecture impair β-adrenergic receptor (BAR) signaling and L-type calcium channel functions, thereby worsening cardiac dysfunction.

Changes in the activity of sarcolemmal enzymes and key ion pumps disrupt ionic homeostasis within cardiac cells^^43^^.

Moreover, cardiac autonomic dysfunction is manifested as impaired baroreflex sensitivity and reduced heart rate variability in advanced cirrhosis, which is a result of persistent activation of the SNS.

Continuous sympathetic stimulation results in prolonged exposure of the myocardium to norepinephrine, and this results in reduced inotropic reserve during stress^^46,47^^.

#### 3.3.2 Inflammatory pathways to reduced myocardial response

Endocannabinoids, particularly anandamide and 2-arachidonoylglycerol, are elevated in cirrhosis. This activates cannabinoid receptor type 1 and blunts BAR- mediated signaling, inhibiting L-type calcium channels and adenylate cyclase, suppressing calcium influx and promoting contractile dysfunction^^43,48^^. Endotoxemia, endocannabinoids, TNF-α, IL-6, and other prominent cytokines impair the microcirculation and facilitate the downregulation of the BAR system, leading to a depressed excitation-contraction coupling of the myocardium^^49,50^^.

#### 3.3.3 Systolic and Diastolic Dysfunction

Left ventricular systolic function in cirrhotic patients often appears to be normal at rest; however, under stress, like exercise or postural changes, the contractile dysfunction becomes more evident. The prevalence of abnormal systolic response is seen in pre-ascitic patients, which includes elevated end-systolic volume and reduced ability to augment ejection fraction during exercise. Echocardiographic studies have confirmed the presence of blunted contractile reserve, increased ventricular filling, and reduced wall compliance in patients during exercise^^43,45^^. At rest, compensatory neurohormonal tone may conceal the systolic weakness. During strenuous situations, impaired BAR function, Ca2+ cycling defect, and limited mitochondrial reserve exhibit blunted stroke volume, rising filling pressure, and pulmonary congestion, therefore expressing the clinical signature of the systolic component in cirrhotic patients^^44,49^^.The abnormality in calcium handling proteins was observed in patients, which includes SERCA2a downregulation, Phospholamban hypophos-phorylation, and ryanodine receptor leakiness, resulting in diastolic calcium overload and impaired lusitropy, facilitating the early diastolic dysfunction observed in CCM^^50^^.Diastolic dysfunction is often considered an early manifestation of CCM and is readily detected by echocardiography at rest, unlike systolic dysfunction^^51^^. Impaired management of calcium and abnormalities in the structural protein of the sarcomere, titin, contribute to the myocardial stiffness and impaired relaxation/lusitropy. The downregulated activity of protein kinase A (PKA), which is essential for the phosphorylation of titin and troponin I, leads to prolonged binding of calcium to troponin C, which delays the relaxation of cardiac muscle, contributing to increased cardiac stiffness^^52^^.In a preclinical experimental study conducted on bile duct ligated (BDL) rats, apart from the downregulation of PKA activity, an increase in collagen-1 expression was noted, which is associated with diastolic stiffness. The experimental activation of RAAS due to reduced effective arterial blood volume, particularly aldosterone, enhances fibrosis by stimulating collagen synthesis by cardiofibroblasts, with this process promoted by an inflammatory cytokine,TGF-β1 (Transforming Growth Factor-beta 1). While these mechanisms are derived from preclinical studies, human data also indicate diastolic dysfunction. Elevated left atrial pressure in cirrhotic patients during stress tests is observed^^50^^. Chronic volume overload driven by hyperdynamic state and neurohormonal activation favors eccentric hypertrophy and interstitial fibrosis. Further MRI examination and tissue data revealed the presence of myocardial edema and fibrosis in advanced cirrhosis, consistent with a diffuse, non-ischemic remodeling pattern^^53^^.

#### 3.3.4 Electrophysiological Disturbances and Role of Bile Acids

**Figure 3:**
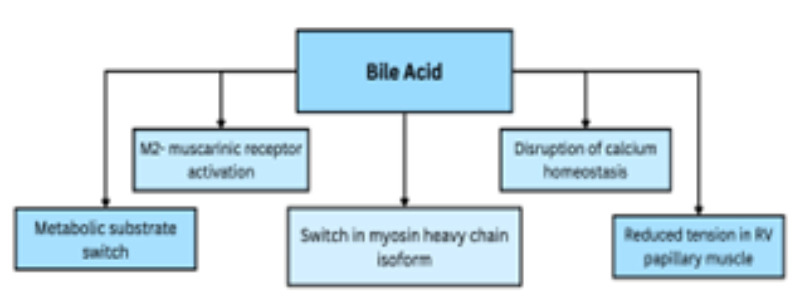
Role of bile acid in the development of CCM in cirrhosis. Exposure of cardiac cells to bile acids causes an inhibitory effect on cardiac contractility. The different mechanisms include i) switch in myosin heavy chain isoform: a rapid switch from A-myosin heavy chain, which is fast contracting, to B-MHC isoform, which is slow contracting, reducing contractility. ii) Disruption of calcium homeostasis: Taurocholate, the product of conjugation of cholic acid with taurine, reduces the calcium transients and impairs sarcoplasmic reticulum calcium release, contributing to reversible suppression of contractility. iii) Reduced tension in RV papillary muscle: under the influence of cholic acid, the amount of tension of right ventricular muscle fiber bundles was significantly less than that in normal conditions. iv) M2 muscarinic receptor activation: Bile acid structurally mimics acetylcholine, which in turn activates M2 receptors, causing a decrease in cAMP, resulting in decreased contractility. v) Metabolic substrate switch: Bile acid has been shown to mediate a switch from fatty acid oxidation to glucose utilization, which is less efficient and results in energy deficit conditions, contributing more to the contractile dysfunction seen in CCM^47,61^. This figure is created using Canva.

QT interval prolongation is frequently observed in cirrhotic patients, which predisposes them to ventricular arrhythmias and sudden cardiac death^^54^^. The underlying includes BAR downregulation, chronic catecholamine exposure, and elevated plasma bile salts. Most insights into the electrophysiological effects of bile acids are derived from experimental models. In a study conducted on BDL rats, it was demonstrated that an increase in serum cholic acid and chenodeoxycholic acid levels is associated with the prolongation of the QT interval, suggesting a potential mechanistic role of bile acids in electrophysiological disturbances^^50,55^^. However, direct causal evidence in humans remains limited, and these findings should be interpreted as hypothesis-generating. Cardiac cells express bile acid receptors, including the nuclear receptor farnesoid X receptor (FXR), although fewer when compared to other tissues. Experimental activation of FXR in cardiomyocytes induces apoptosis through the opening of mitochondrial permeability transition pore^^56^^.Conversely, in a study conducted on BDL rats, experimental reduction of bile acid level was shown to improve systolic and diastolic function^^56^^. **Table 1** summarizes preclinical studies conducted in animal models, highlighting proposed cardiac alterations in cirrhosis, while **Figure 3** summarizes cardiac alterations induced by bile acids in cirrhosis. Clinical evidence indicates that diastolic dysfunction, impaired contractile reserve, and electrophysiological instability collectively drive hemodynamic deterioration and subsequent renal hypoperfusion within the hepato–cardio–renal axis^^57^^. The progressive decline in the ability of the circulatory system to handle the varying hemodynamics triggers a series of neurohumoral cascades, connecting the cirrhotic condition of the liver to the kidney’s hemodynamic disturbances through cardiac intermediary mechanisms.

**Table 1:** Summary of studies conducted on animal models to demonstrate cardiac alterations in cirrhosis This table summarizes preclinical experimental studies using various animal models to examine cardiac alterations in cirrhosis. The studies focus on the observed cardiac changes, underlying mechanisms, and key references for each model, including BDL rats, carbon tetrachloride–treated rats, cholestyramine-treated BDL rats, and cirrhotic mice, which were genetically modified. Each study describes the primary cardiac phenotypes alongside corresponding mechanistic changes derived from animal or in vitro evidence. These findings should be viewed as mechanistic and hypothesis-generated, as direct validation of these pathways in human cirrhosis remains limited or incomplete. Bile-duct Ligation/Ligated (BDL); Left Ventricle (LV); Left Ventricle Diastole/Diastolic Dysfunction (LVDD); β-adrenergic Receptor (BAR); Nitric Oxide (NO); Renin–Angiotensin–Aldosterone System (RAAS); Sympathetic Nervous System (SNS); Farnesoid X Receptor (FXR); Tumor Necrosis Factor-alpha (TNF-α); Double Knockout (DKO); Carbon Tetrachloride (CCl4).

Animal model	Cardiac finding	Observed alterations	Reference
BDL rats	LVDD, chamber dilation, reduced contractile function, prolongation of QT interval	● Increased bile acid levels associated with FXR-mediated cardiomyocytes apoptosis.● Endocannabinoid activation which downregulates BAR responsiveness.● Increase level of NO and Carbon monoxide.● Sarcomere protein alteration.● RAAS overactivation.	^50,55^
CCl4 rat	LV hypertrophy, impaired stress induced contractility	● Chronic hepatic injury associated with over-activation of SNS and RAAS● Fibrosis of liver tissue, collagen disposition● Alteration in Ca2+ handling	^58,59^
BDL+ Cholestyramine reduction	Improved systolic and diastolic function, normalized QT interval	● Experimental reduction of bile acid levels associated with decreased FXR activation.● Restored calcium handling.	^50,60^
Genetic cirrhotic mice (eg. DKO model)	Impaired contractile function, blunted ejection fraction	● Alteration of BAR signaling.● Increased myocardial TNF-α.● Elevated endocannabinoids.	^50^
Isolated cardiomyocytes cirrhotic rats	Reduced L-type Ca2+ channel activity, apoptosis	Prolonged exposure to endocannabinoids and catecholamine resulted in Na+/Ca2+ transport disruption	^58,61^

### 3.4 Hepatorenal Syndrome: Mechanism of Renal Circulatory Alteration

The systemic circulatory changes in the kidney manifested as HRS in advanced liver cirrhosis are from a series of pathophysiologic processes, causing reduced glomerular filtration rate (GFR) and tubular function without any underlying intrinsic renal failure^^62,63^^.

HRS is regarded as functional in most cases, as evident from the kidney transplanted from HRS donors to a non-cirrhotic recipient continuing to function normally; however, extended renal hypoperfusion and systemic inflammation prevalent in cirrhosis can induce ischemic tubular injury, which progresses to acute tubular necrosis^^64^^.

#### 3.4.1 Hemodynamic and Neurohormonal Mechanisms

The manifestation starts when RAAS is activated, along with SNS, as a compensatory response to REABV. The afferent arterioles and juxtaglomerular cells (JGC) contain prorenin, which is converted to renin when JGC senses increased sympathetic tone by BAR or when the macula densa senses decreased delivery of sodium and chloride in the distal convoluted tubule. Then, angiotensinogen (secreted by the liver into circulation) is cleaved by renin to angiotensin I, which is minimally vasoactive^^65^^. Angiotensin II, generated via RAAS activation, promotes renal vasoconstriction, sodium reabsorption, and the release of vasopressin (ADH) and aldosterone, contributing to water retention, reduced renal perfusion, and the development of hepatorenal syndrome^^66^^.

**Figure 4:**
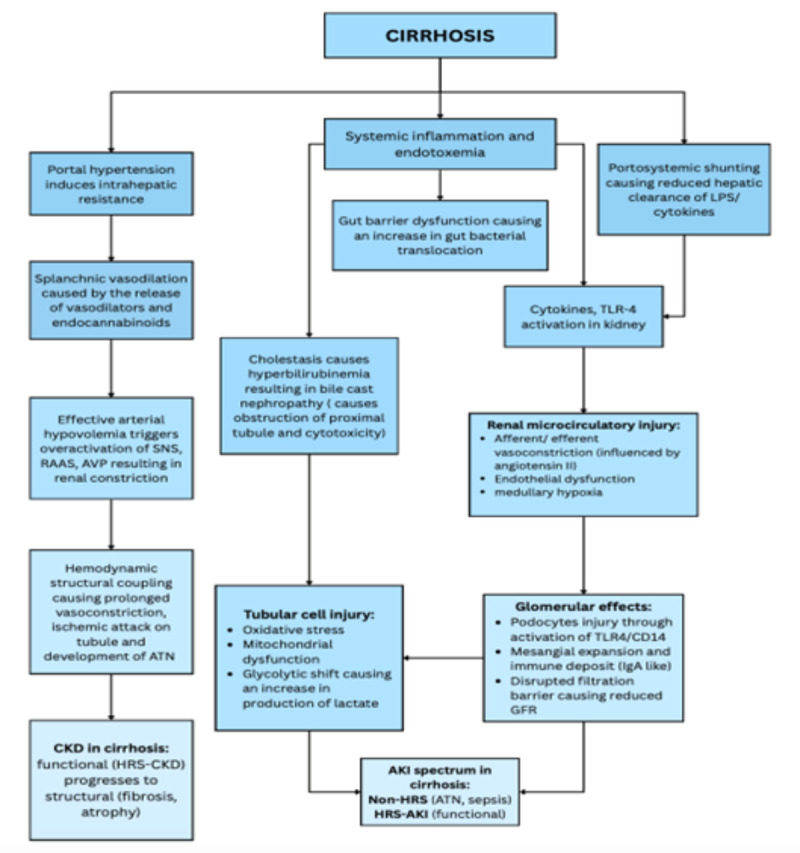
Pathophysiological mechanism of renal dysfunction induced by cirrhosis Splanchnic vasodilation induced by portal hypertension is mediated by vasodilators, which cause a reduction in effective arterial blood volume, stimulating an overactivation of the compensatory neurohormonal activation that is SNS, RAAS, and AVP. Long-term exposure to vasoconstrictors decreases renal blood flow and the glomerular filtration rate (GFR). Ultimately, this may result in renal vasoconstriction along with retention of sodium and water, decreasing the ability to clear free water. All of these events further promote the development of hepatorenal syndrome (HRS). Systemic inflammation and translocation of gut bacteria, and their following processes, worsen the vasoconstriction and microcirculation dysfunction. While structural alterations may coexist, they remain secondary. All these processes collectively cause progressive renal dysfunction^72,73^. This figure is created using Canva.

In addition to the aforementioned process, retention can lead to dilutional hyponatremia, where water retention (manifested as hypervolemia) far exceeds the amount of sodium retained and reabsorbed (manifested as hyponatremia), causing further reduction of overall blood oncotic pressure, one of the factors contributing to ascites^^67,68^^. **Figure 4** below provides a visual summary of the pathophysiological mechanisms of renal dysfunction in cirrhosis.

#### 3.4.2 Endotoxemia and Inflammatory Mechanisms

Another critical factor contributing to HRS in cirrhosis is endotoxemia. Toll-like receptors (TLRs), expressed on hepatic and renal cells, have a major role in the clearance of endotoxins from the circulation. Impairment of this system permits excessive translocation of bacteria beyond the hepatic clearing capacity, aggravating the systemic endotoxemia. Activated neutrophils and inflammatory mediators such as TNFα further aggravate hepatic microcirculation and compromise Kupffer cell function, contributing to reduced clearance^^69^^.

Experimental studies suggest that lipopolysaccharide, along with the other inflammatory responses, causes renal vasoconstriction, reduces tubular flow, and causes podocyte injury through TLR-4 and CD-14 signaling, leading to a decline in GFR^^70^^. Excess concentrations of NO and peroxynitrite formation in experimental models have been associated with renal tubular cell injury, suggesting a mechanistic link to renal dysfunction^^70,71^^. Endotoxemia further worsens tubular injury through TLR4-mediated cytokine release in the S2 and S3 segments of the tubule, causing oliguria and progressive impairment in renal function^^70^^. 

#### 3.4.3 Bile Cast Nephropathy

Bile cast nephropathy is another pathology of the kidney observed in cirrhosis associated with cholestasis. Cholestatic or severe jaundiced cirrhosis presents with increased serum bilirubin, which may lead to bile cast formation in the renal tubule. The hydrophobic bile cast obstructs the renal tubule, causing tubular cytotoxicity, mitochondrial damage, and oxidative stress, resulting in, also known as cholemic nephrosis^^71,74^^. Pathological features of this condition include bile casts in distal tubules, tubular epithelial atrophy, and interstitial inflammation. 

#### 3.4.4 Structural Renal Changes in Cirrhosis

**Figure 5:**
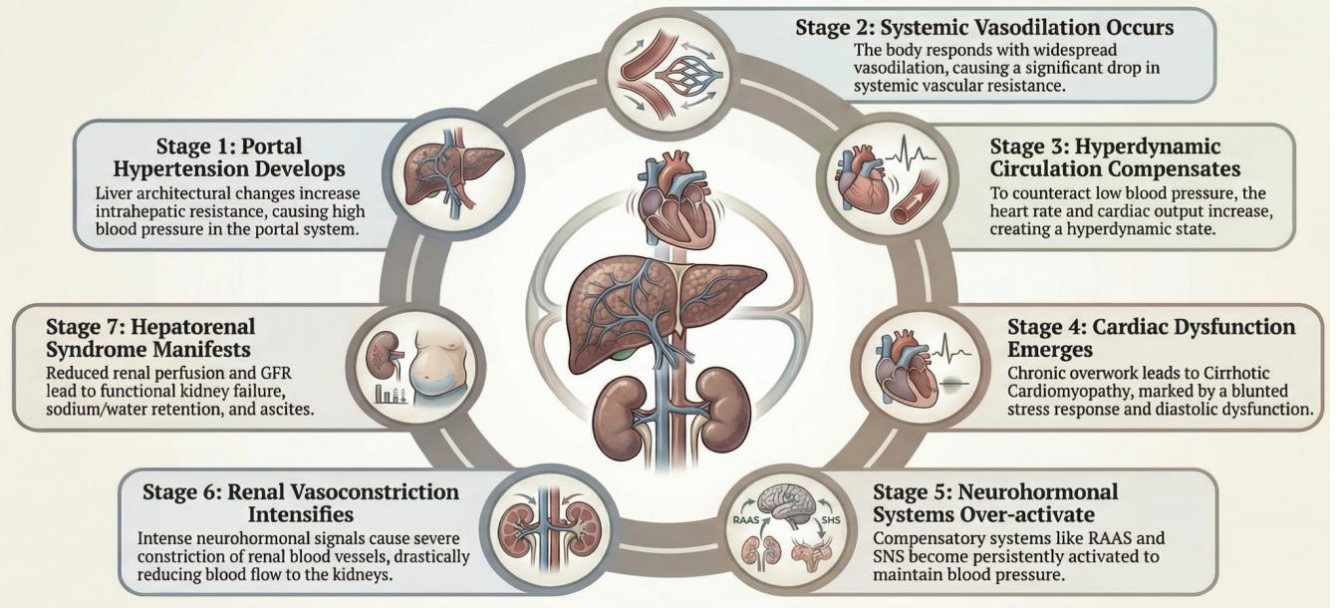
Overview of the Development of Portal Hypertension in Cirrhosis and Associated Hepato-Cardio-Renal Dysfunction The above figure demonstrates the sequential stages of the triad, Hepatocardiorenal axis, which arises because of the cirrhosis-associated portal hypertension leading to systemic vasodilation, hyperdynamic circulation, cardiac dysfunction, renal vasoconstriction, and eventual hepatorenal syndrome. Figure created by the authors using an AI-assisted visualization tool. All components are original and author generated. Not adapted from any previously published source.

Finally, cirrhotic patients may exhibit structural anomalies in the glomeruli, such as mesangial expansion, slight immune complex deposition, arteriolar hyalinosis, tubulointerstitial fibrosis, and lesions resembling IgA nephropathy. Additionally, vascular and tubular structural alterations, including atrophy and fibrosis, were observed, even in the absence of proteinuria or hematuria^^75^^. These changes, when combined with hemodynamic abnormalities and inflammatory mediator activity, can further exacerbate renal impairment and compromise renal perfusion^^76^^.This interconnected cascade of pathophysiologic and hemodynamic changes ultimately accounts for the dysfunction collectively manifested as hepato-cardio-renal syndrome. **Figure 5** below outlines the Pathophysiology of hepatocardiorenal syndrome in Cirrhosis.

## 4. Diagnostic Considerations 

### 4.1 General biomarkers of hepatocardiorenal dysfunction

#### 4.1.1 Cardiac biomarkers

Cardiac biomarkers play a vital role in assessing and managing heart diseases, and they are classified into two categories: molecular and physiological biomarkers.Molecular biomarkers include Atrial natriuretic peptides (ANP) and B-type natriuretic peptides (BNP), along with their prohormone (NT-proBNP). These peptides are released in response to myocardium stretch due to increased preload in cirrhotic conditions by mechanisms, as described earlier, thereby reducing blood pressure. In patients with cirrhosis-related cardiac disease, BNP levels measured at four key time points (T1–T4) have demonstrated 100% sensitivity and 91.9% specificity for detecting cardiac involvement^^44,77,78^^. Another key molecular biomarker, troponin, particularly cardiac troponin T, is elevated in small cardiac lesions, a beneficial biomarker for predicting cardiovascular risk, such as myocardial infarction^^44^^.Physiological biomarkers such as Artificial intelligence-derived cirrhosis electrocardiogram (ACE Score) analysis, echocardiography, Doppler, and ultrasound^^77^^. ACE is an artificial intelligence (AI) model that detects cirrhosis by analyzing the electrocardiogram (ECG). This model gives an ACE

score that helps identify the severity of liver diseases, which is used along with the Model for End-Stage Liver Disease (MELD) and specific laboratory tests. According to clinical studies consisting of 547 patients with cirrhosis, multiple ECGs were obtained before and after liver transplantation to analyze the ACE score. Five years prior to scheduled transplantation, the median ACE score was 0.079. As the cirrhosis progressed, an increase of the ACE score to 0.762 was observed nearing the term of transplantation, followed by a drop to 0.183 within a year after transplantation. Notably, ACE score performance appeared largely independent of commonly used cirrhosis-related medications, such as beta-blockers, diuretics, and lactulose^^79^^.

Several limitations are noted. The limited interpretability of AI models, as the precise ECG features driving predictions are not fully identifiable, which creates challenges for mechanistic understanding and clinical trust ^^79^^. Furthermore, ACE score validation studies are predominantly derived from specialised transplant-centered cohorts, which restricts their applicability to broader cirrhotic groups, particularly compensated disease individuals.

ECG abnormalities observed in patients with CCM include Prolonged QTc interval, lower QRS voltage, and reduced R-R interval variation are observed. Echocardiography, particularly with the aid of tissue Doppler imaging, identifies CCM abnormalities, including hyperdynamic circulation and systolic and diastolic dysfunction^^77,80^^. Ultrasound, along with alpha-fetoprotein blood testing, is a comprehensive diagnostic tool for hepatocellular carcinoma, a critical consideration in cirrhotic patients due to its influence on increasing intrahepatic blood flow resistance. Computed tomography and magnetic resonance imaging serve as alternatives but are often expensive^^81^^.

#### 4.1.2 Portal Hypertension Assessment.

Hepatic Venous Pressure Gradient (HVPG) is a well-established modality in assessing portal hypertension. Normal HVPG is ≤ 5 mmHg^^82^^. An HVPG ≥ 10 mmHG can show the beginning of a decompensated stage. High-risk gastroesophageal variceal bleeding is associated with HVPG ≥ 12 mmHg^^83,84^^.

#### 4.1.3 Renal and urinary biomarkers.

These markers help to indicate kidney function or injury, as well as aid in the early diagnosis and prognosis of kidney disease within the hepatocardiorenal continuum^^62^^. Serum creatinine (SCr) levels in blood increase when renal function declines^^85^^. It is often misjudged in liver cirrhotic patients due to sarcopenia, as it leads to reduced creatinine production, making SCr appear normal even when significant renal impairment is present^^86^^. 

The decreased kidney function leads to microalbuminuria, an early sign of kidney damage. Its increased level indicates a high risk of cardiovascular events^^77,87^^. The early indicators for AKI include neutrophil gelatinase-associated lipocalin (NGAL), interleukin-18 (IL-18), and kidney injury molecule-1 (KIM-1), which reflect tubular injury and inflammatory activation.

Urine NGAL level of 220-250 pg/ml signifies acute tubular necrosis. On the other hand, uNGAL levels for HRS are mildly elevated with 70% improved response to terlipressin (reduced uNGAL), while structural renal injury doesn’t respond to it. Urine NGAL helps to predict acute kidney injury (AKI) 2.5 days earlier compared to SCr and provides the best way to diagnose AKI^^86^^.

#### 4.1.4 Hemodynamic Markers.

Hemodynamic markers integrate cardiovascular and renal physiology. CO is often elevated, along with high serum creatinine, in the early stages of hepatocardiorenal syndrome^^88^^. SVR is often lower due to splanchnic vasodilation, dropping 800 dynes/s/cm⁻⁵/m² ^^89^^.

### 4.2 Serum Biomarkers of Hepatocardiorenal Dysfunction.

Serum biomarkers serve as the most convenient, noninvasive approach in detecting liver damage, inflammation, and early abnormalities within the hepato-cardio-renal continuum.

#### 4.2.1 Liver Injury and Fibrosis Markers.

AST/ALT Ratio (AAR) differentiates the levels of two liver enzymes, aspartate aminotransferase (AST) and alanine aminotransferase (ALT), released in hepatocellular injury^^90^^. Although AAR ≥ 1 indicates cirrhosis in a patient with chronic HCV, numerous studies demonstrate that AAR is not a reliable marker^^91^^. The AST to Platelet Ratio Index (APRI) is a non-invasive marker calculated using a specific formula that is easier and safer compared to other invasive procedures, and it shows much more reliable results than AAR^^91,92^^. 

#### 4.2.2 Inflammatory and Immune Activation Markers.

Systemic inflammatory markers show the progression of hepatocardiorenal dysfunction. Immune markers such as IL-6 and Growth Differentiation Factor (GDF-15) are high in patients with worsening liver cirrhosis^^93^^. Moreover, TNF-α levels are also elevated, a major apoptotic cytokine for hepatic cells in cirrhotic conditions^^94^^.Portosystemic shunt development and disruption in gastrointestinal barrier integrity can reveal bacterial DNA and endotoxins to the systemic circulation, which indicates bacterial translocation^^95^^. Increased levels of endothelial active substances such as NO, ROS, and endothelin 1 are also notable^^27^^. 

#### 4.2.3 Acute Phase Reactants.

**Figure 6:**
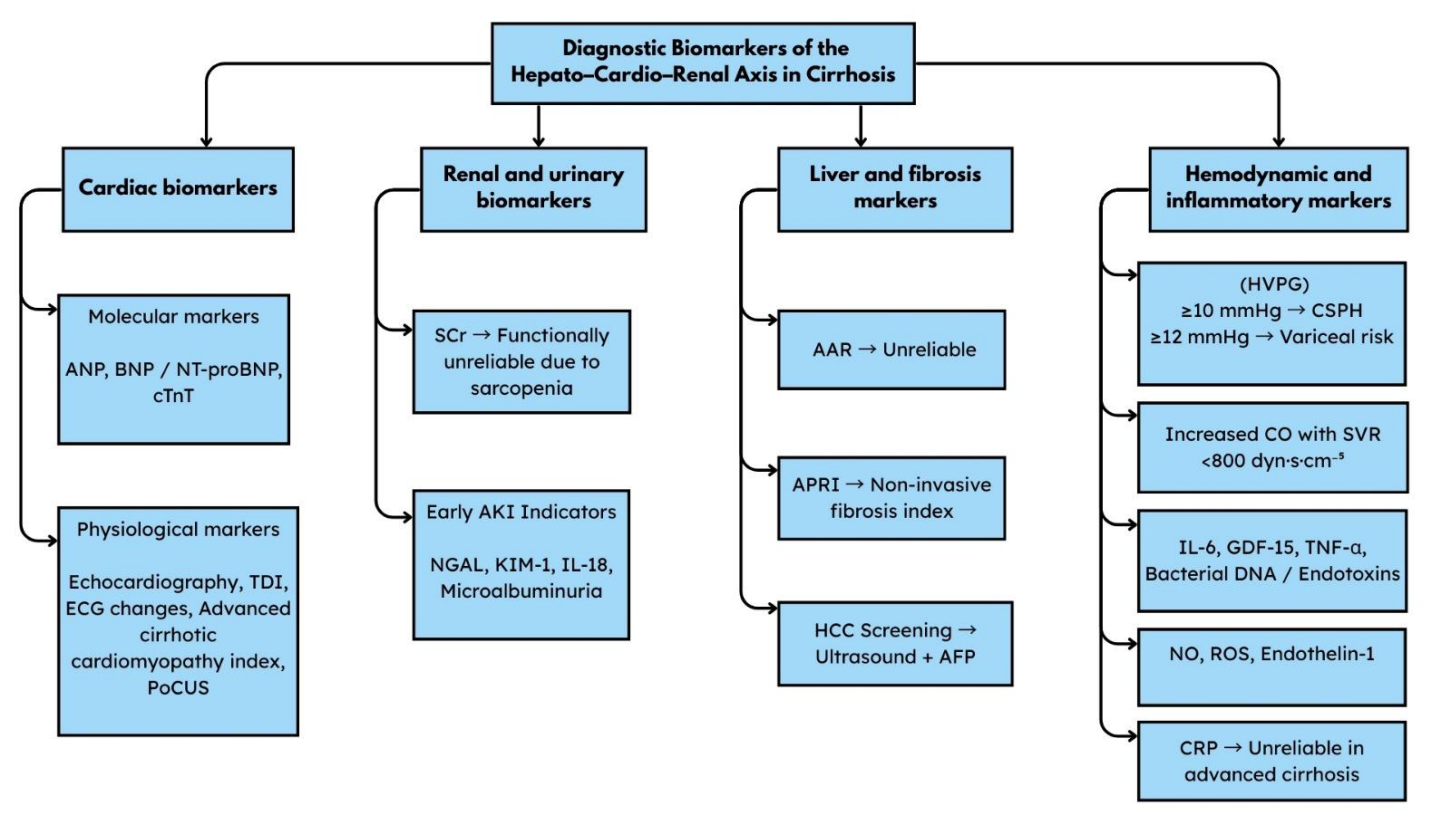
Major diagnostic biomarkers of the hepato–cardio–renal axis in cirrhosis This figure is created using Canva. Artificial intelligence–cirrhosis–electrocardiogram (ACE); Alpha-fetoprotein (AFP); Aspartate aminotransferase to alanine aminotransferase ratio(AAR); Atrial natriuretic peptide (ANP); Aspartate aminotransferase to platelet ratio index (APRI); Acute kidney injury (AKI); B-type natriuretic peptide (BNP); Cardiac troponin T (cTnT ); Cardiac output (CO); C-reactive protein (CRP); Clinically significant portal hypertension (CSPH); Electrocardiogram (ECG); Growth differentiation factor-15 (GDF-15); Hepatocellular carcinoma (HCC); Hepatic venous pressure gradient (HVPG); Interleukin-6 (IL-6); Interleukin-18(IL-18); Kidney injury molecule-1 (KIM-1); Model for End-Stage Liver Disease (MELD); Neutrophil gelatinase-associated lipocalin (NGAL); Nitric oxide (NO); N-terminal pro-B-type natriuretic peptide (NT-proBNP); Point-of-care ultrasound (PoCUS); Reactive oxygen species (ROS); Serum creatinine (SCr); Systemic vascular resistance (SVR); Tissue Doppler imaging (TDI0); Tumor necrosis factor-alpha (TNF-α).

Although C-reactive protein is higher at the beginning of cirrhosis due to systemic inflammation, as the condition progresses, the liver's synthetic ability wanes, and the level of CRP lowers even when infection is noted. Due to this paradox, CRP cannot be considered a reliable marker^^96^^. **Figure 6** summarizes major cardiac, renal, urinary, liver, and various hemodynamic and inflammatory biomarkers involved in the hepato-cardiac-renal axis in cirrhosis.

## 5. Therapeutic Horizon

The therapeutic landscape helps us to restore the hemodynamic dysfunction that arises along the hepato-renal-cardiac axis. Current pharmacological and non-pharmacological approaches restore the effective arterial circulation, improve the CO, and

renal perfusion. **Table 2** shows an overview of drugs used for various therapeutic applications, their mechanism of action, along their toxicities.

### 5.1 Pharmacological

Vasoconstrictors with albumin are preferred first-line therapy for hepatorenal dysfunction, as they increase the mean arterial pressure, decrease serum creatinine, and increase urinary output by improving renal perfusion and subsequently reducing the activation of RAAS, sympathetic nervous system, and arginine vasopressin as they counteract the primary hemodynamic abnormality of the axis^^97,98^^. Terlipressin, a vasopressin analog, is the most widely used and preferred first-line vasoconstrictor^^97^^. Randomized controlled trial showed continuous intravenous infusion has fewer side effects and is more effective at a lower dosage than it being administered by intravenous (IV) bolus^^99^^. Furthermore, its effectiveness of treatment is poor when administered alone without albumin^^100^^. Second-line vasoconstrictor regimens include norepinephrine or the combination of midodrine and octreotide plus albumin, used when IV terlipressin therapy is unavailable or contraindicated. Studies and controlled trials show that terlipressin or norepinephrine, plus albumin, has better efficacy than Midodrine/Octreotide plus albumin, as the Terlipressin, a vasopressin analog, is the most widely used and preferred first-line vasoconstrictor^^97^^. Randomized controlled trial showed continuous intravenous infusion has fewer side effects and is more effective at a lower dosage than it being administered by intravenous (IV) bolus^^99^^. Furthermore, its effectiveness of treatment is poor when administered alone without albumin^^100^^. Second-line vasoconstrictor regimens include norepinephrine or the combination of midodrine and octreotide plus albumin, used when IV terlipressin therapy is unavailable or contraindicated. Studies and controlled trials show that terlipressin or norepinephrine, plus albumin, has better efficacy than Midodrine/Octreotide plus albumin, as the group that was administered terlipressin had a significantly higher mean arterial pressure and the reduction of serum creatinine was higher too^^98,101^^.Another pharmacological treatment is diuretics, which serves as supportive therapy for volume overload through sodium and water excretion and increasing the urine production. Thiazide diuretics should be avoided when the creatinine level is too high^^102^^. Chlorthalidone, a thiazide-like diuretic, can be used to treat hypertension in stage 4 of chronic kidney disease (CKD), but on the other hand, it carries side effects like hyponatremia, reduced GFR, which cause AKI, therefore requires careful monitoring^^103,104^^. Metolazone, another thiazide-like diuretic, can be used in combination with loop diuretics for decompensation of congestive heart failure and severe refractory congestive heart failure ^^105,106^^. Spironolactone, a potassium-sparing mineralocorticoid receptor antagonist, has adverse effects like gynecomastia and hyperkalemia, which are especially more common in Asian patients, therefore necessitating careful monitoring ^^107^^. Sodium-glucose cotransporter 2 (SGLT2) inhibitors like dapagliflozin and empagliflozin exert cardio-renal protective adjunct therapies, exerting antioxidant and anti-fibrotic effects, antioxidant effects and anti-fibrotic effect, can stimulate the synthesis of AMP-activated protein kinase, can regulate inflammatory responses^^108,109^^. These diverse effects serve as useful add-ons for addressing the cardiac and renal dysfunction in cirrhotic patients. Angiotensin receptor neprilysin inhibitor (ARNI), sacubitril (neprilysin inhibitor)/valsartan (angiotensin II receptor blocker) can be used to treat patients with severe HFrEF. In a cohort, comparing perindopril (ACE inhibitor) and ARNI showed that left ventricular ejection fraction (LVEF) was improved by 11.8% and 11.5% respectively^^110^^. It is worth noting that a case of hepatotoxicity associated with sacubitril/valsartan has been reported^^111^^. Lastly, non-selective beta-blockers (NSBBs) like carvedilol can be used for the prevention of variceal hemorrhage and the management of portal hypertension. Long periods of use can reduce the risk of decompensation of cirrhosis, portal hypertension, as well as secondary infections^^112,113^^.

**Table 2:** Therapeutic Applications and Pharmacologic Profiles of Current Treatment Regimens The table above summarizes drugs used for each clinical abnormality and their mechanisms of action. Prolonged use or high dosages of these drugs may be associated with drug-related toxicity. MAP: mean arterial pressure, GFR: glomerular filtration rate, AKI: Acute kidney injury, MRA: mineralocorticosteroids, UTI: urinary tract infection, NSBBs: nonselective beta-blockers, HFrEF: heart failure with reduced ejection fraction, ARNI: angiotensin receptor neprilysin inhibitor.

Therapeutic Application	Drug Name/Regimen	Primary Mechanism of Action	Toxicities	Contraindications	Reference
Hepatorenal Dysfunction	Vasoconstrictors + Albumin - Terlipressin, Norepinephrine, Midodrine/Octreotide	Increases MAP and improves renal perfusion.	Terlipressin: Fewer side effects with continuous IV infusion compared to IV bolus.	Severe hypoxia, respiratory compromise.	^97-101^
Fluid/Volume Overload	Thiazide-like diuretic - Chlorthalidone, Metolazone	Promotes sodium and water excretion.	Risks: hyponatremia, reduced GFR, and AKI.	High creatinine level	^102-106^
	Spironolactone (MRA)	Controls volume overload (potassium-sparing).	Gynecomastia and Hyperkalemia (common in Asian patients)	Hyperkalemia	^107^
Portal Hypertension/Variceal Hemorrhage	NSBBs - Carvedilol	Reduces portal pressure.	Hypotension, Inadequate kidney perfusion	Refractory ascites	^112-114^
HFrEF	(ARNI) (Sacubitril/Valsartan)	Blocks Angiotensin II receptor and inhibits neprilysin.	Hepatotoxicity	Biliary cirrhosis, Cholestasis	^110,111^
Cardio-Renal Protection	SGLT2 inhibitors - Dapagliflozin, Empagliflozin	Antioxidant, anti-fibrotic, and regulatory effects.	Hypotension, UTI	Decompensated cirrhosis (without monitoring)	^108,109,115^

### 5.2 Non-Pharmacological

Non-pharmacological therapeutic approaches include liver transplantation, transjugular intrahepatic portosystemic shunt (TIPS), and kidney replacement therapy, each having its own advantages and disadvantages. Liver transplantation is the definitive and best curative therapeutic option, because it can eliminate the root cause and reverse kidney damage, and restore the splanchnic circulation, but it is effective in about 83% of patients^^116^^. TIPS implantation serves as an important bridge in reducing portal hypertension by side-to-side shunt between the portal and hepatic vein and is considered a treatment of choice in patients with liver cirrhosis^^117^^. TIPS is now used for many other complications of portal hypertension, such as refractory ascites (RA), hepatic hydrothorax, HRS, non-tumoral portal vein thrombosis, and hepatopulmonary syndrome. Currently, expanded-polytetrafluoroethylene (e-PTFE) stents are used for TIPS, which are much more reliable and less prone to dysfunctions^^118^^.KRT (kidney replacement therapy) serves as supportive rescue therapy in diuretic-resistant patients to promote decongestion and decompensated heart failure. If the patient is on vasoconstrictors plus albumin, then KRT will provide no significant improvement and might lead to longer hospital stays^^119^^. Extracorporeal ultrafiltration is beneficial for acute decompensated heart failure, as it can reverse the volume overload, whereas peritoneal dialysis, a continuous form of KRT, is better suited for chronic refractory heart failure and can remove the excess sodium^^120^^.

### 5.3 Lifestyle and nutritional interventions

Daily caloric intake of 30-35 kcal/kg is recommended along with a restriction on intake of sodium to 2 g/day in case of no response to diuretics and consumption of 1.2-1.5 g of protein/kg/day to prevent loss of muscle. Patients should prioritize complex carbohydrates, consume healthy, small, frequent meals, and include a healthy late evening snack to prevent the hypermetabolic state. Zinc, potassium, magnesium, and phosphorus supplements should be considered if a patient is administered diuretics. Vitamins A, B1, B2, B6, B9, B12, D, and E supplements are required. These vitamins are often deficient due to malabsorption and impaired liver function. Alcohol should be avoided, as it could cause more damage to the liver^^121,122^^. Folic acid deficiency is often seen in patients with decompensated alcohol-related liver cirrhosis^^123^^. Folate deficiency (FD) can cause hyperhomocysteinemia, a central driver of oxidative stress, endothelial injury, and hepatic inflammation, and adequate intake of folate supplementation can reduce homocysteine level^^124^^. Furthermore, FD disrupts lipid metabolism and impairs the DNA methylation to hallmarks for NAFLD and ALD progression. However, excessive folate is also associated with HCC. Since both deficiency and excessive amounts of folate can be destructive, maintaining balanced levels is essential^^8^^. In a retrospective study, patients prescribed with folic acid at discharge showed a 50% lower liver-transplantation risk and decreased hospital readmission^^121^^.

## 6. Challenges and Limitations

A major barrier arises from the persistence of fragmented and organ-centric definitions, as they are still diagnosed under separate umbrellas despite the evidence of overlapping pathophysiological mechanisms^^124^^. For reference, common cardiac diagnostic thresholds, such as reduced cardiac ejection fraction or prolonged E/A ratios, often fail to identify the subtle dysfunction seen in CCM. Although evolving criteria involving global longitudinal strain (GLS) and advanced diastolic parameters have been proposed, they are not yet standardized and remain inconsistent. Similarly, HRS definitions are limited by their reliance on serum creatinine level; given muscle wasting and altered metabolism, it can be considered an unreliable metric in cirrhosis^^124^^. Emerging biomarkers like urinary NGAL or KIM-1 seem promising in detecting renal injury in cirrhosis. However, most evidence comes from small, single-center studies with limited external validation, and no standardized threshold exists to distinguish vasodilation-dominant from congestion-dominant injury. This diagnostic ambiguity further contributes to the frequent misclassification rate^^125^^. Notably, point-of-care ultrasound (PoCUS) helped to demonstrate that many presumed cases of HRS-AKI were due to venous congestion rather than hypoperfusion. Nevertheless, PoCUS protocol remains unsystematized, operator-dependent, and underutilized in clinical practice. Cost and accessibility further limit their access, emphasizing the need for standardization and validation^^9^^.In terms of therapeutic interventions, terlipressin combined with albumin remains the most widely studied therapy for HRS-AKI. Meta-analyses reveal that it doubles HRS reversal rates and normalizes serum creatinine levels. However, it raises a serious risk of respiratory failure and is contraindicated in patients with predispositions like volume overload, pulmonary hypertension, and right-sided strain. The absence of robust predictive tools for treatment responsiveness forces clinicians to make precarious decisions. Alternative regimens, like norepinephrine or the midodrine-octreotide combination, are less variably used and lack standardization, with minimal direct comparison or guidance^^10,11^^. Another interventional strategy, the TIPS, further contributes to therapeutic complexity. While TIPS can improve renal perfusion and fluid management, it may trigger or unmask subclinical cardiac dysfunction; almost 18-20% of cirrhotic patients who underwent TIPS developed post-TIPS decompensation linked to diastolic pathology, which includes pathological E/A ratios, elevated E/e, cardiomegaly, or elevated pulmonary artery systolic pressure (PASP). This significantly reduces the overall survival rate^^12^^.Clinical trials in HRS-AKI often concentrate on short-term markers, such as HRS reversal or reductions in serum creatinine level, while neglecting clinically meaningful outcomes like transplant-free survival, longer-term renal recovery, prevention of recurrence of AKI, or quality of life. Many studies often exclude patients with cardiac-related disorders, advanced acute-on-chronic liver failure (ACLF), or pulmonary hypertension, even though these patients require special attention^^12^^. This selective approach limits the generalizability of current therapies. Rarely, hepatologists, cardiologists, and nephrologists consider integrated multi-organ assessments. Investigations that simultaneously evaluate advanced imaging methods like speckle-tracking echocardiography and cardiac MRI and PoCUS, kidney biomarkers, and liver severity scores within the same patient population remain rare. This lack of integrated assessment hinders both mechanistic understanding and the practical translation across clinical specialties^^13^^.Global inequities pose a hindrance to the implementation of best practices. Many advanced and useful diagnostics, like speckle strain echocardiography, PoCUS, NGAL or IL-18 assays, and even interventions like TIPS, are unavailable or unaffordable in low- and middle-income regions, where the burden of cirrhosis is highest. These limitations exacerbate inequities in early detection, monitoring, and therapeutic management. Consequently, restricting the real-world impact of these promising tools. Even the availability of terlipressin remains restricted globally, constraining uniform therapy implementation^^126^^. The overall effect is a healthcare system that is divided, reactive, and focused on one single organ, rather than coordinated, forward-looking, and attentive to the patient as a whole. Thereby constraining our ability to recognize and manage the complexity of the interactions between liver, heart, and kidney in cirrhosis^^13^^.Liver transplantation adds further complexity as MELD-based allocation fails to detect cardiac reserve and renal reversibility. Subclinical CCM and HRS often remain unrecognized until the transplant stress reveals circulatory collapse or delayed renal recovery. The absence of reliable tools to distinguish reversible HRS from intrinsic renal disease, along with challenges in simultaneous liver-kidney transplant decisions and perioperative hemodynamic management, remains a major determinant of outcomes^^127^^.

## 7. Future directions

### 7.1 Integrated Axis-Aware Diagnostics

Future integrated, axis-aware staging systems should be considered; this helps to replace more fragmented definitions currently in use. Such a framework would enable more precise diagnoses and tailored treatment strategies, which improve outcomes for patients with triad syndrome^^128,129^^. In addition, early tubular injury could be detected through biomarkers such as urinary NGAL and KIM-1, interpreted against cirrhosis-specific thresholds. This helps to distinguish structural damage from hemodynamic dysfunction and supports timely adjustments to enhance therapeutic efficacy^^130^^.Multi-omics approaches, including proteomics, metabolomics, and transcriptomics, may help to delineate vasodilation-dominant HRS from congestion-driven cardiorenal dysfunction by identifying novel biomarkers and molecular signatures; these diagnostic advances could help shift towards precision therapy^^131^^. In cases where transplantation is indicated, axis-aware transplant pathways have to be considered that include structured pre-LT cardiac assessment that incorporates strain, right-sided pressures, and congestion images, as well as pre-LT renal assessment using cystatin C and urinary biomarkers to forecast post-transplant kidney and heart outcomes^^132^^. Looking forward, exploring smart devices like sensor-equipped stents and catheters that can monitor pressures in the liver and heart in real time after TIPS. These devices could help physicians in early detection of abnormality in circulation, allowing timely adjustments in care, and preventing kidney and heart problems from getting worse^^133,134^^. 

### 7.2 Genetic, molecular, and cellular regeneration approaches

Future innovation may consider using stem cell therapies, like mesenchymal and muse cells, to repair the liver, heart, and kidney cells simultaneously. In preclinical animal studies, muse cells have been observed to differentiate into various cell types like hepatocytes, cholangiocytes, Kupffer cells, and podocytes in a study done on animals. This suggests a possibility in regenerating multiple organs affected by cirrhosis^^135,136^^. Another notable approach tested in animal models of early heart or kidney injury associated with cirrhosis focused on mitochondria, which improved energy function and reduced tissue damage caused by lowered blood flow and reperfusion. These effects have not been demonstrated in humans^^137,138^^.At the molecular level, the use of taurine supplementation has shown promise in protecting the heart in cirrhosis in animal studies. These studies reported a reduction in oxidative stress and support of mitochondrial function, suggesting potential benefits for heart-kidney interactions ^^139^^.Emerging treatments include genetic and cell-based therapies as an option for cirrhosis. One promising approach uses regenerative macrophage therapy, which mainly targets fibrogenic and immune pathways, especially those driven by TGF-β, to preserve organ function. These strategies could help restore and address the multi-organ complications of cirrhosis^^140^^. 

### 7.3 Microbiome modulation

Microbiome modulation is another emerging area. Studies using probiotics, synbiotics, and fecal microbiota transplantation have shown improvement in liver function. It also helped in reducing ammonia levels, fewer episodes of hepatic encephalopathy, and lower systemic toxins in patients. Moreover, application of this has been shown to increase short-chain fatty acids, which produce taxa, improve barrier function, and attenuate hyperdynamic circulation in cirrhosis. These findings also suggest that balancing gut bacteria could help reduce inflammation, improve circulation, and ease stress on the kidney^^141^^. 

### 7.4 Non-pharmacologic and extracorporeal support

Nonpharmacologic innovations include improvement in extracorporeal liver support. Although older albumin dialysis machines and bioartificial systems have shown modest benefits, research has shown that devices like advanced cartridges, sorbents, and cellular bioreactors are designed to lower the strain on circulation and provide patients with short-term support while they wait for the organ allocation in complex cases of cirrhosis^^142^^.Further studies are being done in this field so that specific venous Doppler patterns can improve kidney outcome without causing hypotension or reduced liver blood flow^^143^^. 

### 7.5 AI, technology, and future predictive tools

Wearable devices that could detect early pulmonary congestion or early drops in blood pressure are likely to become part of cirrhosis care. This could prompt timely medication changes or clinical visits^^144^^. Integrating artificial intelligence and machine learning can make use of clinical, imaging, and biomarker data^^145^^. Future AI tools must be explainable rather than a black box. Clinicians must be aware of the factors that are driving the patient’s rising risk score so they can respond with the right intervention^^146^^. Therefore, AI can help predict when a patient is at risk of decompensation, guide physicians in adjusting treatments, and prevent hospitalizations by identifying any rising risk^^147^^.

## 8. Conclusion

Liver cirrhosis causes a multisystemic cascade of pathophysiologic processes, collectively manifesting as hepato-cardio-renal syndrome. Common manifestations are portal hypertension, splanchnic vasodilation and hypervolemia, endotoxemia, systemic inflammation, hyperdynamic circulation, ascites, variceal bleeding, CCM, and HRS. The utilization of multi-modal biomarkers and imaging tools integrated with clinical scoring and AI enhances early detection, diagnosis, and management of cardiorenal dysfunction in liver cirrhosis. It includes various cardiac biomarkers, physiological biomarkers, renal and urinary biomarkers, various direct and indirect serum biomarkers, immunological markers, hemodynamic markers (especially HVPG), and usage of general liver cirrhosis prognostic markers such as MELD. The treatment is a combination of pharmacological and non-pharmacological interventions and lifestyle modifications. The pharmacological approach includes the usage of vasoconstrictors, more prominently, terlipressin administered with albumin. Others include diuretics, SGLT-2 inhibitors, ARNI, and NSBB. A prominent approach for non-pharmacological treatment remains liver transplantation. Other approaches include TIPS and KRT. Lifestyle modifications include vitamin supplementation and dietary modification to prevent a hypermetabolic state.As for management, it requires an axis-aware staging system consisting of integrated personalized care with the coordination of respective specialists. In the future, the usage of point-of-care systems and the current therapeutic innovations in the fields of stem cells, genetics, biochemistry, and microbiology, along with the technological advancements in wearables and AI, have shown promising results.

## BULLET POINTS

**·** **Cirrhosis is a multisystem disorder that affects especially the heart and kidney via “Hepatocardiorenal axis’’, characterized by portal hypertension, splanchnic vasodilation, systemic inflammation, neurohormonal activation.**

**·** **Mechanisms of cirrhotic cardiomyopathy (CCM) involve impaired BAR signaling, altered membrane fluidity, ion-channel dysfunction, and neurohormonal changes.**

**·** **Modern diagnostic tools include biomarkers and AI-derived instruments.**

**·** **Emerging stem cell therapies, mitochondrial target strategies, microbiome therapies, and extacorporeal support system advancements can be considered promising options for improving treatment outcomes.**

## Open questions

1. Is there a need for an integrated, axis-aware diagnostic test that simultaneously evaluates liver, cardiac, and renal function in cirrhosis, rather than assessing each organ separately?

2. What is the long-term potential of emerging therapies—regenerative, mitochondrial, and microbiome-based—to prevent or reverse liver–heart–kidney dysfunction in cirrhosis?

3. How can advanced diagnostics and therapies (e.g., HVPG, NGAL/KIM-1, TIPS, terlipressin) be adapted for effective use in resource-limited settings with a high cirrhosis burden?

4. In high-risk cohorts with latent CCM, ACLF, borderline cardiac reserve, how can we estimate treatment responsiveness to various pharmacological drugs such as terlipressin, norepinephrine, TIPS, NSBBs, ARNI, and SGLT2 inhibitors?

5. What is the ideal timing for TIPS and for liver transplantation, and what practical approach can help physicians assess renal reversibility and cardiac vulnerability when considering the need for combined liver-kidney transplantation?

6. Can simple bedside tools like poCUS venous doppler assessments and strain echocardiography be standardized for detecting early congestion, guiding fluid and vasoconstrictor treatment, and also avoiding mistakes in diagnosing HRS-AKI?

7. What are the safest and most effective ways to integrate AI into clinical practice while using ECG patterns, poCUS images, strain data, biomarkers, and clinical information and at the same time maintaining transparency, trustworthiness, regulatory expectations, and clinical confidence?
